# Differential use of antidiabetic medication related to income, cohabitation and area of residence: a Swedish nationwide cohort study

**DOI:** 10.1136/jech-2024-223262

**Published:** 2025-02-26

**Authors:** Paulina Jonéus, Björn Pasternak, Ingvild Odsbu, Carolyn E Cesta, Rino Bellocco, Ylva Trolle Lagerros, Laura Pazzagli

**Affiliations:** 1 Clinical Epidemiology Division, Department of Medicine Solna, Karolinska Institutet, Stockholm, Sweden; 2 Department of Epidemiology Research, Statens Serum Institut, Copenhagen, Denmark; 3 Department of Chronic Diseases, Norwegian Institute of Public Health, Oslo, Norway; 4 Centre for Pharmacoepidemiology, Department of Medicine Solna, Karolinska Institutet, Stockholm, Sweden; 5 Università degli Studi di Milano-Bicocca, Milano, Italy; 6 Department of Medical Epidemiology and Biostatistics, Karolinska Institutet, Stockholm, Sweden; 7 Department of Medicine Huddinge, Karolinska Institutet, Stockholm, Sweden

**Keywords:** DRUG PRESCRIPTIONS, DIABETES MELLITUS, Health inequalities, LONGITUDINAL STUDIES, PHARMACOEPIDEMIOLOGY

## Abstract

**Background:**

Poor adherence to antidiabetic medication in individuals with type 2 diabetes (T2D) may lead to increased risk of morbidity and mortality. Socioeconomic and demographic factors associated with non-adherence have been mainly identified via cross-sectional studies. To investigate the association between antidiabetic medication adherence and income, cohabitation and area of residence.

**Methods:**

Register-based cohort study of individuals with T2D living in Sweden and initiating antidiabetic treatment between 2006 and 2022. Confounding adjustment and competing events were accounted for via inverse probability of treatment and censoring weighting. Exposures were disposable income, social income support, cohabitation status and area of residence. Outcomes were antidiabetic medication adherence during the first year from treatment initiation and treatment interruption at 12 and 60 months.

**Results:**

This study included 594 918 individuals with T2D. Low disposable income (adjusted OR: 1.18, 95% CI: (1.14 to 1.21)), social income support (1.09, (1.05 to 1.14)), living in large cities (1.28, (1.24 to 1.31)) and cohabitation (1.09, (1.06 to 1.11)) were associated with non-adherence (proportion of days covered ≤0.2), as compared with high adherence (proportion of days covered >0.8). Consistently, treatment interruption was associated with social income support (relative risk ratio at 12 months: 1.10, (1.06 to 1.14) and at 60 months 1.02 (1.00 to 1.05)), living in large cities (1.13, (1.12 to 1.14); 1.08, (1.07 to 1.08)) and low income (1.05, (1.03 to 1.07); 1.01, (1.00 to 1.02)).

**Conclusions:**

Low income levels, cohabitation and living in large cities were associated with non-adherence to antidiabetic medication and risk of treatment interruption. The results highlight the need for targeted interventions aiming at improving adherence to treatments both at patient and healthcare system levels.

**WHAT IS ALREADY KNOWN ON THIS TOPIC**

**WHAT THIS STUDY ADDS**

- This large cohort study, including 594 918 individuals with type 2 diabetes (T2D) living in Sweden who initiated treatment between 2006 and 2022, used advanced epidemiological and statistical methods to investigate the association between antidiabetic medication adherence, including treatment interruption and social income support, individual disposable income, cohabitation status and area of residence.

**HOW THIS STUDY MIGHT AFFECT RESEARCH, PRACTICE OR POLICY**

- This study found social income support, low income levels, cohabitation and living in large cities to be associated with non-adherence to antidiabetic medications and treatment interruption, suggesting that a composite of modifiable socioeconomic and demographic factors influences adherence to treatments for T2D. Closer monitoring of risk groups, improvement of access to healthcare facilities, increase of financial aid in deprived settings, patient education in health management and healthcare practitioner training in treatment management, are all potential actions that could be planned aiming at the reduction of long-term consequences of T2D.

## Introduction

 The management of type 2 diabetes (T2D) involves a variety of individualised treatment strategies. Adherence to treatment recommendations is essential to decrease the risk of diabetes-related comorbidities. Still, poor adherence is common and has consequences, such as an increased risk of morbidity and premature mortality.^1^,^3^ Improving treatment management and adherence are crucial clinical and public health priorities to prevent long-term complications in individuals with T2D.

A wide range of socioeconomic factors associated with antidiabetic medication adherence was presented in a recent global systematic overview.^4^ Antidiabetic medication adherence was associated with older age, male sex, higher education, higher income and urban living. The few large cohort studies were, however, restricted to only one type of antidiabetic medication, or excluded insulins, and analyses were limited to selected groups of individuals, for example, only those found in a single pharmacy database.^5^,^9^ This might lead to the loss of information on individuals with more severe T2D, as well as loss of socioeconomic heterogeneity and consequently lack of generalisability. These gaps in knowledge on socioeconomic and demographic factors related to different aspects of medication adherence highlight the need for large nationwide studies including individuals with complex treatment patterns.

Driven by this research gap, this cohort study used nationwide data from Sweden and advanced epidemiological and statistical methods to investigate the association between antidiabetic medication adherence, including treatment interruption and income, cohabitation status and area of residence in individuals with T2D.

## Methods

### Study design and data sources

This is a non-interventional, register-based cohort study of individuals with T2D identified in the Swedish national health and population registers and the Swedish National Diabetes Register (NDR). The Prescribed Drug Register (PDR) includes information on all prescriptions filled at the pharmacies, including the Anatomical Therapeutic Chemical (ATC) classification codes and the amount dispensed. Socioeconomic and demographic variables were retrieved from the longitudinal integrated database for health insurance and labour market studies (LISA), and from the Total Population Register. The National Patient Register contains information on all inpatient and outpatient specialist care visits with diagnoses (International Classification of Diseases codes, ICD-10), date of admission and discharge. The NDR has had good coverage since 2014 (88% in 2019) and provided additional information on clinically confirmed T2D diagnoses.^10^

### Study population

To identify individuals with T2D, the source population included all new users of antidiabetic medication who initiated treatment with a non-insulin glucose-lowering drug (ATC A10B) between 1 July 2006 and 30 September 2022, allowing for a wash-out period of 1 year from the launch of the PDR (1 July 2005). When evaluating adherence during the first year from treatment initiation, the population was additionally restricted to individuals with a follow-up of at least 1 year before death, migration or administrative end of study (30 September 2022). All individuals lacking information on any of the exposure variables were excluded from the analyses. Flowcharts of the study populations are presented in online supplemental figure 1.

### Exposures

Four main exposure variables were used as proxies to capture the individual’s socioeconomic and demographic position: area of residence, disposable income, presence of income support and cohabitation status, measured in the year before treatment initiation (detailed definitions in online supplemental table 1). For area of residence, the Swedish municipalities were categorised according to the classification provided by the Swedish Association of Local Authorities and Regions as large cities, medium-sized towns or smaller towns and rural municipalities. All individuals in the study population were categorised depending on quartiles of disposable income per consumption unit in the year before treatment initiation.^11^ The presence of social income support indicates if there had been any family financial aid during the year to assist individuals in managing their basic needs. Lastly, cohabitation was defined as living with a married or registered partner, or having someone else in the household contributing to the disposable income.

### Outcomes

Two primary outcomes were investigated: adherence to antidiabetic medication regimens during the first year and risk of interruption of antidiabetic medication at 12 and 60 months from treatment initiation. When constructing treatment episodes, individuals were allowed to switch between and add different treatments (including insulins) to the initial therapy during follow-up, since adherence to any antidiabetic medication, including switching and add-ons, is of clinical relevance.^10^

All antidiabetic treatments were classified into seven drug classes: insulins, biguanides (metformin), sulfonylureas, dipeptidyl peptidase 4 inhibitors, glucagon-like peptide 1 receptor agonists (GLP-1a), sodium-glucose cotransporter 2 inhibitors and others (online supplemental table 2). Multiple concurrent dispensations of different drug classes were allowed, and the dispensed classes determined the assumed treatment regimen. A dispensed drug from a new class was defined as an add-on if the current treatment/s received were refilled within 60 days after introducing the additional one and the dispensations of the current and the new treatment overlapped for at least 60 days, and as a switching otherwise.^12^ Drugs from different classes dispensed on the same day were assumed to be taken concurrently. Both add-ons and switching to a new drug were considered from the date the prescription of the new drug was filled.

Adherence to the antidiabetic medication regimens during the first year after treatment initiation was measured with the proportion of days covered (PDC) by treatments,^13^ corrected for days hospitalised. For this outcome, four PDC levels were considered, where PDC≤0.2 indicated non-adherence to antidiabetic medication, 0.2<PDC≤0.5 low adherence, 0.5<PDC≤0.8 moderate adherence and PDC>0.8 high adherence.

Treatment interruption was defined as the first interruption of all antidiabetic medication from treatment initiation, considering death as a competing event and administrative end of study, emigration or hospitalisation for more than 60 days as censoring events. This outcome corresponded to the duration of the first treatment episode (of continuous use) of any antidiabetic medication regimen. An interruption was defined as the first time a treatment regimen was not refilled within a predefined allowable gap of 90 days.^14 15^

### Background covariates and potential confounders

Background covariates were included to account for other health and background differences (online supplemental table 3). To refrain from adjusting for covariates that occurred after exposure, all covariates were measured the year prior to the measurement of the exposure variables (online supplemental figure 2). The number of inpatient and outpatient specialist visits (proxies for health consumption), cardiovascular diseases, depression, obesity and hypertension measured via diagnoses and prescription fills, together with the Charlson Comorbidity Index adapted to register-based research in Sweden to account for overall morbidities (online supplemental table 4), were included.^16^ In addition, the number of other prescriptions filled (number of unique fifth level ATC codes), as a proxy for polypharmacy, sex, age, education and country of birth were also included.

### Statistical analyses

Evaluating adherence to the antidiabetic medication regimens, the association between exposures and PDC during the first year was estimated with a multinomial regression model. The outcome model included all four exposure variables, and the adjustment for background covariates and potential confounders was done via inverse probability of treatment weighting (IPTW), where the exposure variables were modelled as functions of the background covariates and potential confounders to construct the individuals’ weights (Text S1).^17^

When estimating the risk of interruption, the cumulative incidence curves, quantifying the exposure effect on the risk of first treatment interruption of all antidiabetic medication, not mediated by the competing event of death, were calculated.^18^ Adjustment for background covariates and potential confounders was done via IPTW. Logistic regression models pooled over time were used to model the observed cause-specific risk of antidiabetic medication interruptions, where the different competing and censoring events (death, hospitalisation, emigration and administrative end of study) were modelled separately with inverse probability of censoring weighting (IPCW) (Text S1). Relative risk ratios were estimated and bootstrapping with 1000 replicates was used to compute the CIs.

### Supplementary analyses

When evaluating adherence to the antidiabetic medication regimens, the individual’s country of birth was used to perform subgroup analyses. In addition, subgroup analyses were done including individuals in the study population initiating antidiabetic medication before and after the beginning of the COVID-19 pandemic. Finally, the analysis was repeated in the subpopulation of individuals with a clinically confirmed T2D diagnosis recorded in the NDR. Informed by results from the first outcome analysis, relative risk ratios of interruption at 12 and 60 months comparing a high-risk group to a low-risk group were estimated in an exploratory analysis.^19^

## Results

### Adherence during the first year from treatment initiation

The analytical population consisted of 498 187 individuals (online supplemental figure 1) with mean age of 61.4 years and including 44.1% women (table 1). In total, 9.9% of the study population was classified as non-adherent, 40.1% had low adherence, 31.4% moderate and 18.6% high. Online supplemental figure 3 shows the mean absolute standardised differences for all included covariates before and after IPTW weighting (summary statistics of IPTW weights in online supplemental table 5). Weighted mean differences and SDs for background covariates in the four outcome groups are presented in online supplemental table 6.

**Table 1:** Exposure variables, background covariates and potential confounders for individuals included in the analyses of adherence measured with the proportion of days covered (PDC) the first year after treatment initiation and time to first treatment interruption

Study populations	Analysis of adherence measured with the PDC the first year after treatment initiation	Analysis of time to first treatment interruption
Exposure variables		
Place of residence		
Large cities (%)	31.46	31.7
Medium-sized towns (%)	40.96	40.85
Small towns (%)	27.58	27.45
Unknown (%)	0	0
Disposable family income per consumption unit		
Mean and SD	2461.68 (5207.83)	2292.67 (10 249.07)
Unknown (%)	0	0
Presence of income support		
No (%)	94.29	94.35
Yes (%)	5.71	5.65
Unknown (%)	0	0
Cohabitation status		
Yes (%)	61.73	61.2
No (%)	38.27	38.8
Unknown (%)	0	0
Background covariates and potential confounders		
Age (mean and SD)	61.42 (14.40)	62.02 (14.71)
Migration (%)	0.86	0.84
Sex, female (%)	44.16	44.16
County of birth		
Sweden (%)	76.08	76.14
The Nordic countries excl. Sweden (%)	4.4	4.39
Europe excl. EU27 and the Nordic countries (%)	4.39	4.31
EU27 excl. the Nordic countries (%)	3.38	3.39
Asia (%)	8.09	8.12
Africa (%)	2.33	2.34
North America (%)	0.3	0.3
South America (%)	0.91	0.88
Oceania (%)	0.02	0.02
Unknown (%)	0.1	0.11
Education		
Less than secondary education (%)	30.83	30.53
Secondary education (%)	44.87	44.67
More than secondary education (%)	22.29	22.65
Unknown (%)	2	2.15
Unemployed for more than 6 months		
No (%)	96.62	96.74
Yes (%)	3.26	3.11
Unknown (%)	0.12	0.15
Main income source		
Employment (%)	42.32	41.35
Unemployment (%)	1.02	0.97
Early retirement and social security (%)	15.76	15.18
Old age pensions (%)	38.68	40.27
None (%)	2.02	2
Unknown (%)	0.2	0.23
CCI score		
None (CCI score of 0) (%)	93.45	92.6
Moderate (CCI score of 1–4) (%)	6.35	7.13
Severe (CCI score ≥5) (%)	0.2	0.27
Number of other filled prescriptions		
Mean and SD	5.59 (5.21)	5.75 (5.30)
CVD (%)	5.3	6.09
Depression (%)	14.66	15
Hypertension (%)	51.79	52.97
Obesity (%)	1.55	1.46
Number of impatient care days		
>20 and ≤50 (%)	0.93	1.02
>0 and ≤10 (%)	10.06	10.36
>10 and ≤20 (%)	1.37	1.49
0 (%)	87.29	86.76
>50 (%)	0.35	0.36

CCI, Charlson Comorbidity Index; CVD, cardiovascular diseases; EU27, European Union including the 27-member countries, income variables are in multiples of 100 SEK; PDC, proportion of days covered.

Results from the multinomial IPTW weighted regression model (table 2) showed that non-adherence (PDC≤0.2) compared with high adherence (PDC>0.8) was associated with living in large cities as compared with smaller (adjusted OR (aOR): 1.28, 95% CI: (1.24 to 1.31)), cohabitation (1.09, (1.06 to 1.11)), having low disposable income as compared with high (1.18, (1.14 to 1.21)) and receiving social income support (1.09, (1.05 to 1.14)). Moderate adherence compared with high adherence was associated with living in smaller towns compared with larger cities. Both low and moderate adherence, compared with high adherence, were associated with not receiving social income support.

**Table 2:** Associations between area of residence, cohabitation, income and social income support and proportion of days covered (PDC) by any antidiabetic medication in the first year after treatment initiation

	N	Non-adherent (PDC≤0.2) vs high adherence (PDC>0.8)		Low adherence (0.2<PDC≤0.5) vs high adherence (PDC>0.8)		Moderate adherence (0.5<PDC≤0.8) vs high adherence (PDC>0.8)	
		aOR	95 % CI	aOR	95 % CI	aOR	95 % CI
Area of residence							
Large cities	156 749	1.28	1.24 to 1.31	0.99	0.97 to 1.01	0.88	0.87 to 0.90
Medium-sized towns	204 055	0.95	0.93 to 0.98	0.86	0.85 to 0.88	0.90	0.89 to 0.92
Smaller towns	137 383	Ref		Ref		Ref	
Cohabitation							
Yes	307 536	1.09	1.06 to 1.11	1.05	1.04 to 1.07	1.04	1.02 to 1.05
No	190 651	Ref		Ref		Ref	
Income							
Income quartile 1 (lowest)	124 407	1.18	1.14 to 1.21	1.10	1.07 to 1.12	1.21	1.19 to 1.24
Income quartile 2	124 573	1.07	1.04 to 1.10	1.09	1.06 to 1.11	1.16	1.13 to 1.19
Income quartile 3	124 584	1.02	0.99 to 1.05	1.04	1.01 to 1.06	1.09	1.06 to 1.11
Income quartile 4	124 623	Ref		Ref		Ref	
Social income support							
Yes	28 448	1.09	1.05 to 1.14	0.92	0.89 to 0.95	0.94	0.91 to 0.97
No	469 739	Ref		Ref		Ref	

aOR, adjusted OR; CI, confidence interval; N, total number of individuals per exposure group; PDC, proportion of days covered.

The majority, 80.5%, of the study population was born in the Nordic countries, followed by 8.1% in Asia, and 11.4% in other or unknown countries. For individuals from the Nordic countries and Asia, non-adherence compared with high adherence showed similar results as for the main analysis. For individuals from other or unknown countries, cohabitation showed an aOR of 0.93 (0.87 to 0.99), and having low disposable income, as compared with high, an aOR of 1.30 (1.19 to 1.41) (table 3).

**Table 3:** Associations between area of residence, cohabitation, income and social income support and proportion of days covered (PDC) by any antidiabetic medication in the first year after treatment initiation, by country of birth

	N	Non-adherent (PDC≤0.2) vs high adherence (PDC>0.8)		Low adherence (0.2<PDC≤0.5) vs high adherence (PDC>0.8)		Moderate adherence (0.5<PDC≤0.8) vs high adherence (PDC>0.8)	
		aOR	95% CI	aOR	95% CI	aOR	95% CI
**Individuals born in the Nordic countries**							
Area of residence							
Large cities	107 406	1.25	1.21 to 1.29	0.99	0.97 to 1.01	0.89	0.87 to 0.91
Medium-sized towns	169 080	0.91	0.89 to 0.94	0.84	0.83 to 0.86	0.89	0.87 to 0.91
Smaller towns	124 481	Ref		Ref		Ref	
Cohabitation							
Yes	237 164	1.07	1.04 to 1.10	1.02	1.00 to 1.04	1.02	1.00 to 1.04
No	163 803	Ref		Ref		Ref	
Income							
Income quartile 1 (lowest)	81 825	1.14	1.10 to 1.19	1.07	1.05 to 1.10	1.20	1.17 to 1.23
Income quartile 2	102 845	1.04	1.01 to 1.08	1.07	1.04 to 1.09	1.16	1.13 to 1.19
Income quartile 3	105 717	1.00	0.97 to 1.03	1.03	1.00 to 1.05	1.08	1.05 to 1.11
Income quartile 4	110 580	Ref		Ref		Ref	
Social income support							
Yes	9010	1.14	1.08 to 1.19	0.92	0.89 to 0.95	0.95	0.92 to 0.98
No	391 957	Ref		Ref		Ref	
**Individuals born in Asia**							
Area of residence							
Large cities	20 092	1.30	1.18 to 1.42	0.99	0.92 to 1.06	0.81	0.75 to 0.88
Medium-sized towns	15 372	1.19	1.09 to 1.30	0.98	0.92 to 1.06	0.88	0.82 to 0.95
Smaller towns	4847	Ref		Ref		Ref	
Cohabitation							
Yes	31 062	1.16	1.08 to 1.25	1.16	1.09 to 1.23	1.11	1.04 to 1.18
No	9249	Ref		Ref		Ref	
Income							
Income quartile 1 (lowest)	20 317	1.11	1.00 to 1.23	1.05	0.96 to 1.14	1.21	1.10 to 1.32
Income quartile 2	8144	1.03	0.94 to 1.14	1.04	0.96 to 1.13	1.12	1.03 to 1.22
Income quartile 3	6874	1.07	0.98 to 1.18	1.02	0.94 to 1.10	1.12	1.03 to 1.21
Income quartile 4	4976	Ref		Ref		Ref	
Social income support							
Yes	10 776	1.13	0.95 to 1.34	1.05	0.91 to 1.21	0.94	0.81 to 1.10
No	29 535	Ref		Ref		Ref	
**Individuals born in other or unknown countries**							
Area of residence							
Large cities	29 251	1.18	1.10 to 1.27	0.96	0.90 to 1.01	0.88	0.82 to 0.94
Medium-sized towns	19 603	1.00	0.93 to 1.08	0.91	0.86 to 0.97	1.04	0.98 to 1.10
Smaller towns	8055	Ref		Ref		Ref	
Cohabitation							
Yes	39 310	0.93	0.87 to 0.99	1.10	1.05 to 1.16	1.11	1.05 to 1.17
No	17 599	Ref		Ref		Ref	
Income							
Income quartile 1 (lowest)	22 265	1.30	1.19 to 1.41	1.24	1.16 to 1.32	1.29	1.20 to 1.39
Income quartile 2	13 584	1.15	1.06 to 1.25	1.19	1.12 to 1.27	1.21	1.13 to 1.30
Income quartile 3	11 993	1.03	0.95 to 1.11	1.08	1.02 to 1.15	1.13	1.05 to 1.20
Income quartile 4	9067	Ref		Ref		Ref	
Social income support							
Yes	8662	1.10	0.96 to 1.26	1.03	0.93 to 1.15	0.89	0.79 to 1.00
No	48 247	Ref		Ref		Ref	

aOR, adjusted OR; CI, confidence interval; N, total number of individuals per exposure group; PDC, proportion of days covered.

Most individuals, 76.9%, initiated treatment before the COVID-19 pandemic. Non-adherence (compared with high) was associated with living in large cities as compared with smaller for individuals in both the pre-COVID-19 and post-COVID-19 groups (1.23, (1.20 to 1.28) and 1.38, (1.31 to 1.46)), cohabitation (1.06, 95% CI: (1.03 to 1.09) and 1.17, (1.12 to 1.22)), and having low disposable income (1.08, (1.04 to 1.13) and 1.43, (1.34 to 1.53)) (table 4).

**Table 4:** Associations between area of residence, cohabitation, income and social income support and proportion of days covered (PDC) by any antidiabetic medication in the first year after treatment initiation, pre-COVID-19 and post-COVID-19 pandemic

	N	Non-adherent (PDC≤0.2) vs high adherence (PDC>0.8)		Low adherence (0.2<PDC≤0.5) vs high adherence (PDC>0.8)		Moderate adherence (0.5<PDC≤0.8) vs high adherence (PDC>0.8)	
		aOR	95 % CI	aOR	95 % CI	aOR	95 % CI
Treatment initiation pre-COVID-19							
Area of residence							
Large cities	118 761	1.23	1.20 to 1.28	0.95	0.93 to 0.98	0.87	0.85 to 0.90
Medium-sized towns	156 416	0.95	0.92 to 0.98	0.86	0.85 to 0.88	0.92	0.90 to 0.94
Smaller towns	107 743	Ref		Ref		Ref	
Cohabitation							
Yes	236 891	1.06	1.03 to 1.09	1.03	1.01 to 1.05	1.02	1.00 to 1.04
No	146 029	Ref		Ref		Ref	
Income							
Income quartile 1 (lowest)	106 316	1.08	1.04 to 1.13	1.01	0.99 to 1.04	1.10	1.07 to 1.13
Income quartile 2	98 067	1.00	0.97 to 1.04	1.01	0.99 to 1.04	1.09	1.06 to 1.12
Income quartile 3	94 459	0.96	0.93 to 1.00	0.99	0.97 to 1.02	1.05	1.02 to 1.08
Income quartile 4	84 078	Ref		Ref		Ref	
Social income support							
Yes	21 736	1.16	1.10 to 1.22	0.93	0.90 to 0.97	0.94	0.90 to 0.97
No	361 184						
Treatment initiation post-COVID-19							
Area of residence							
Large cities	37 988	1.38	1.31 to 1.46	1.11	1.06 to 1.15	0.89	0.85 to 0.92
Medium-sized towns	47 639	0.95	0.90 to 1.00	0.86	0.83 to 0.89	0.86	0.82 to 0.89
Smaller towns	29 640	Ref		Ref		Ref	
Cohabitation							
Yes	70 645	1.17	1.12 to 1.22	1.09	1.05 to 1.12	1.02	0.99 to 1.06
No	44 622	Ref		Ref		Ref	
Income							
Income quartile 1 (lowest)	18 091	1.43	1.34 to 1.53	1.12	1.07 to 1.18	1.10	1.04 to 1.16
Income quartile 2	26 506	1.22	1.15 to 1.29	1.14	1.09 to 1.19	1.04	0.99 to 1.09
Income quartile 3	30 125	1.14	1.09 to 1.20	1.07	1.03 to 1.10	1.04	1.00 to 1.08
Income quartile 4	40 545	Ref		Ref		Ref	
Social income support							
Yes	6712	0.89	0.82 to 0.98	0.93	0.87 to 0.99	1.02	0.95 to 1.10
No	108 555	Ref		Ref		Ref	

aOR, adjusted OR; CI, confidence interval; N, total number of individuals per exposure group; PDC, proportion of days covered.

The subanalysis with the study population restricted to 331 261 individuals with a clinically confirmed T2D diagnosis showed an association between non-adherence and living in large cities as compared with smaller (1.23, (1.19 to 1.28)), having a low disposable income (1.25, (1.19 to 1.30)) and receiving social income support (1.31, (1.24 to 1.39)). Non-adherence was not associated with cohabitation (online supplemental table 7).

### Time to first interruption of all antidiabetic medication

In the analysis for the outcome of the first interruption of all antidiabetic medication from treatment initiation, 22 439 individuals (3.8%) were removed due to missing data on any of the exposure variables. Among these, 20 134 individuals had a registered migration to and/or from Sweden, and 9534 individuals immigrated to Sweden in the year of measuring the exposures. Excluded individuals had a mean age of 49.7 years and 46.0% were women, 54.0% had missing place of residence and 60.9% had missing information on disposable family income, income support and cohabitation status (online supplemental table 8). The majority was born in Asia (48.9%), followed by Africa (14.7%) and Sweden (14.6%). A high proportion had missing information on education (98.3%) and employment status (98.2%).

The final analytical population consisted of 572 479 individuals, among whom 35.5% had a treatment interruption during the first 12 months and 58.0% during the follow-up of 60 months.

There were a few extreme values in the IPCW and IPTW weights (online supplemental table 9). Online supplemental figures 4,5 show the mean absolute standardised differences for all included covariates before and after IPTW-IPCW weighting at 12 and 60 months from treatment initiation.

**Figure 1:**
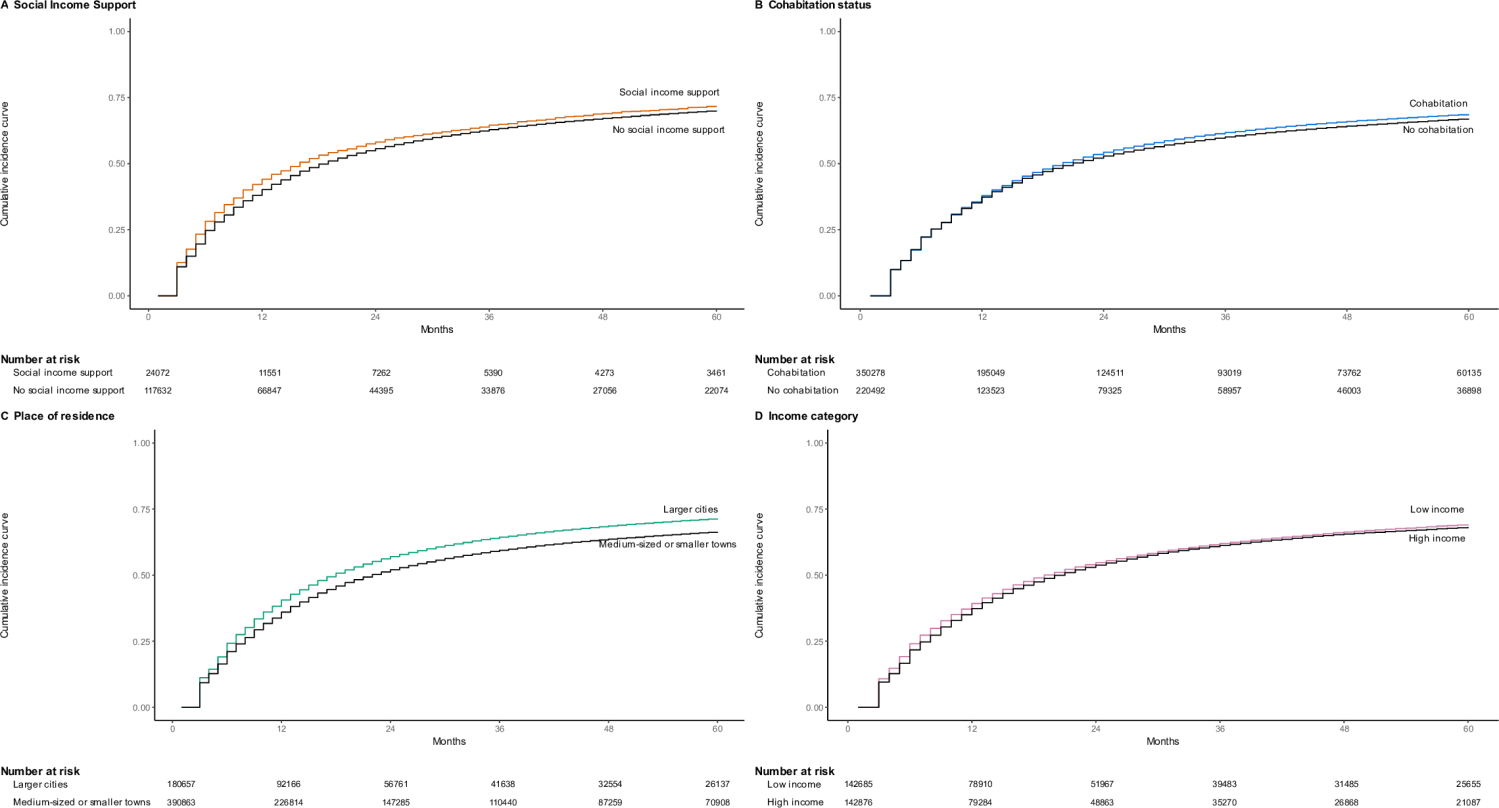
Cumulative incidence curves for antidiabetic medication interruption, up until 60 months after treatment initiation, for the different exposure variables.

Weighted cumulative incidence curves for treatment interruption for each of the four exposure groups are presented in figure 1. Interruption was associated with receiving social income support (relative risk ratio at 12 months: 1.10, 95% CI: (1.06 to 1.14) and at 60 months 1.02, 95% CI: (1.00 to 1.05)), living in large cities as compared with smaller or medium-sized towns (1.13, (1.12 to 1.14); 1.08, (1.07 to 1.08)), cohabiting (1.01, (1.01 to 1.02); 1.02, (1.02 to 1.03)) and low income as compared with high (1.05, (1.03 to 1.07); 1.01, (1.00 to 1.02)).

Informed by results from the first outcome analysis, individuals living in a large city, having low income with social income support and cohabitating formed a high-risk group. Compared with this reference group, living alone with a high income in a small town was associated with a reduced risk of interruption at 12 months (0.62, (0.46 to 0.89)) and 60 months (0.87, (0.75 to 1.05)).

## Discussion

In this nationwide cohort study, low income levels, cohabitation and living in large cities were associated with non-adherence to antidiabetic medication and risks of treatment interruption in individuals with T2D. These results suggest that a composite of socioeconomic and demographic factors influences adherence to treatments for T2D. Recent public health literature suggests considering these factors as modifiable, like many other risk factors for chronic diseases.^20 21^ Together with closer monitoring of risk groups, the improvement of access to healthcare facilities, increase of financial aid in deprived settings, patient education in health management and healthcare practitioner training in treatment management, are all potential actions that could be planned for health improvement and reduction of long-term consequences of T2D.

In Sweden, there is high-cost protection for healthcare visits and prescribed drugs, where adults pay a maximum of Kr1400 (US$128, 5 December 2024) for outpatient visits, and Kr2850 (US$262, 5 December 2024) for medication in a 12-month period.^22^ However, a recent survey among individuals with diabetes showed that 15% had difficulties in managing costs related to their diagnosis and, in some cases, refrained from healthcare visits and medication.^23^ Another study showed that there may be socioeconomic and demographic disparities in access to GLP-1a as well as geographical differences in clinical interpretation of treatment guidelines.^24^ In addition, disparities in healthcare consumption are influenced by the ability to understand and apply health information, social inequality in health as a result of, for example, living conditions and poor social relations and other demographic factors.^25^ Previous cohort studies, limited by restriction to one geographical area, a small study population or a subgroup of antidiabetic treatment alternatives, reported associations between adherence to antidiabetic medication and income levels in line with our study.^5 6 9^ An increase in the odds of non-adherence was found in individuals born outside of the Nordic countries and Asia, having low disposable income, as compared with high. These results are in line with earlier findings showing lower adherence in minorities and individuals with lower socioeconomic status.^7 26 27^

Living in large cities compared with smaller towns increased the odds of non-adherence compared with high adherence. In addition, treatment interruption was also associated with living in large cities. There are no prior studies comparing adherence to antidiabetic medication between different residential areas in Sweden; nonetheless, large regional differences in healthcare provided and prescription patterns were previously found.^24 28 29^ For other types of medication, discontinuation has been found to be positively associated with living in larger cities.^30^ A cohort study in the USA found an association with higher adherence to antidiabetic medication when living in rural compared with urban regions, in line with the results in this study.^7^

An increase in the odds of non-adherence was also found for cohabitation. Previous studies assessing the association between adherence to antidiabetic medication and civil status showed inconsistent results.^4 9^ Another Swedish study investigating discontinuation of insulin during pregnancy showed a not statistically significant association for women not cohabiting with a partner and discontinuation.^31^ Additionally, several studies reported that being married may increase adherence levels.^4^ However, the definition of cohabitation used in the current study does not consider only marital status, and a larger household may lead to the need for sharing the family disposable income among several family members, and may impair the possibility of coping with one’s health issues.

In March 2020, COVID-19 restrictions were imposed limiting the possibilities of physical meetings for monitoring T2D within primary care and for collecting prescribed medications at pharmacies leading to changed patterns of prescription dispensation.^32^ In this study, an increase in the odds of non-adherence was found in both the pre-COVID-19 and post-COVID-19 groups for cohabitation, living in large cities and low income, which is in line with the main analysis.

Strengths of this study include the large size of the population-based cohort and the use of national Swedish registers which provide extensive data coverage, with no risk of recall bias. The PDR ensures coverage of all dispensed drugs in Sweden. By linkage to socioeconomic data, which includes a large set of administrative register data and to the National Patient Register, where all inpatient and outpatient specialist care visits are recorded, this study could evaluate associations between socioeconomic position and adherence to antidiabetic medication. In addition, the current work relied on the use of advanced epidemiological and statistical methods aiming to address confounding and selection biases. The study population was based on new users of blood glucose-lowering drugs, which thereby did not include prevalent users, which could have affected the definition of adherence. Finally, when defining adherence, this study was not limited to the initially prescribed drug nor a fixed number of antidiabetic medications, instead all individuals were allowed to switch between and add different treatments to the initial therapy during follow-up. This is of clinical importance since the management of T2D involves a variety of individualised antidiabetic therapies, and new treatment alternatives were introduced in recent years.

Among the limitations, there were missing values in the registers due to, for example, recent immigration. Exclusion of individuals with missing information on socioeconomic and/or demographic exposures may limit the generalisability of the findings. Descriptive information on excluded individuals showed that the majority were born outside of Sweden, had a mean age of 49.7 years and 46.0% were women. Another limitation is that it was not possible to account for primary non-adherence to prescribed medication, as the data only included records on filled prescriptions. In addition, using filled prescriptions measures, the study could identify only refill behaviour and not actual medication intake, nevertheless, a continuous refill pattern could be considered as a good proxy for it. Specific reasons for non-adherence or interruption were not explored in this study; remission in T2D is, however, rare.^33^ Finally, the main study population was identified by drug initiation, although a supplementary analysis of the population with a clinically confirmed diagnosis of T2D showed aligned results and confirmed robustness of the definition of the study population.

In conclusion, the findings of this study revealed that low income levels, cohabitation and living in large cities do have an association with adherence levels and treatment interruption. From a public health perspective, these factors are potentially modifiable.

These results highlight the need for targeted interventions aiming at improving adherence to treatments for T2D both at patient and healthcare system levels. The findings also raise questions regarding equitable access to care. Despite the fact that Sweden has universal health coverage, the study found a differential use of antidiabetic medication in relation to socioeconomic and demographic factors. These differences should be reduced in a healthcare system which provides equal care to all individuals suggesting that interventions may also be directed to improve access to care. Results from this study also indicate that future research should focus on under-investigated populations such as, for example, immigrants, who may have substantial problems accessing healthcare and consequently also adhering to treatments. Poor adherence to treatments may lead to long-term complications of T2D, and the role of adherence as a mediator along the pathway between treatment and long-term complications, is key and should be considered when planning individual treatment strategies.

## Supplementary material

10.1136/jech-2024-223262 online supplemental file 1

## Data Availability

No data are available.
