# Supplementary material for: Differential use of antidiabetic medication related to income, cohabitation and area of residence: a Swedish nationwide cohort study
Source: J Epidemiol Community Health. 2025 Feb 26;79(8):e223262. doi: 10.1136/jech-2024-223262 (PMC12322451; doi:10.1136/jech-2024-223262)
Supplement: online supplemental file 1 [file jech-79-8-s001.pdf]

SUPPLEMENTARY MATERIAL

Differential use of antidiabetic medication related to income, cohabitation and area of residence: a Swedish nationwide cohort study

Paulina Jonéus[1], PhD; Björn Pasternak[1,2], MD, PhD; Ingvild Odsbu[3], PhD; Carolyn E Cesta[4], PhD; Rino Bellocco[5,6], PhD; Ylva Trolle Lagerros[7], MD, PhD; Laura Pazzagli[1,*], PhD;

Affiliations

[1] Clinical Epidemiology Division, Department of Medicine Solna, Karolinska Institutet, Stockholm, Sweden
[2] Department of Epidemiology Research, Statens Serum Institut, Copenhagen, Denmark
[3] Department of Chronic Diseases, Norwegian Institute of Public Health, Oslo, Norway
[4] Centre for Pharmacoepidemiology, Karolinska Institutet, Stockholm, Sweden
[5] Department of Statistics and Quantitative Methods, University of Milano-Bicocca, Milano, Italy
[6] Department of Medical Epidemiology and Biostatistics, Karolinska Institutet, Stockholm, Sweden
[7] Department of Medicine Huddinge, Karolinska Institutet, Stockholm, Sweden

[*] **Corresponding author**: Laura Pazzagli, Clinical Epidemiology Division, Department of Medicine Solna, Karolinska Institutet, 171 76 Stockholm, Sweden. Email: laura.pazzagli@ki.se. https://orcid.org/0000-0002-1908-6073

Running title: Differential use of antidiabetic medication

Contents

Figure S1: Flow-chart of study populations for the two outcomes ... 3

Table S1: Definitions of the exposure variables ... 4

Table S2: Included antidiabetic medication, ATC codes, assumed daily doses and calculation of duration of the prescriptions ... 5

Table S3: Definitions of the background covariates and confounding variables ... 6

Figure S2: DAG representing relationships between outcome, exposures and background covariates, including confounders ... 7

Table S4: The different comorbidity domains included in the Charlson comorbidity index (CCI) adapted to a Swedish setting ... 8

Text S1: Subject-specific stabilized weight calculation ... 9

Figure S3: Mean absolute covariate balance at baseline, analysis of adherence measured with the PDC during the first year from treatment initiation ... 10

Table S5: Summary statistics of IPTW weights, analysis of adherence measured with the PDC during the first year from treatment initiation ... 11

Table S6: Summary statistics in the four outcome groups, analysis of adherence measured with the PDC during the first year from treatment initiation ... 12

Table S7: Subgroup analysis evaluating adherence via the PDC the first year after treatment initiation, subpopulation with a registered T2D diagnosis in the National Diabetes Register ... 13

Table S8. Exposure variables, and some background covariates and potential confounders for individuals excluded from the analysis of time to first treatment interruption of all antidiabetic medication ... 14

Table S9: Summary statistics of IPTW-IPCW weights, analysis of the outcome first interruption of all antidiabetic medication ... 15

Figure S4: Mean absolute covariate balance at 12 months, analysis of the outcome first interruption of all antidiabetic medication ... 16

Figure S5: Mean absolute covariate balance at 60 months, analysis of the outcome first interruption of all antidiabetic medication ... 17

References ... 18

Figure S1: Flow-chart of study populations for the two outcomes

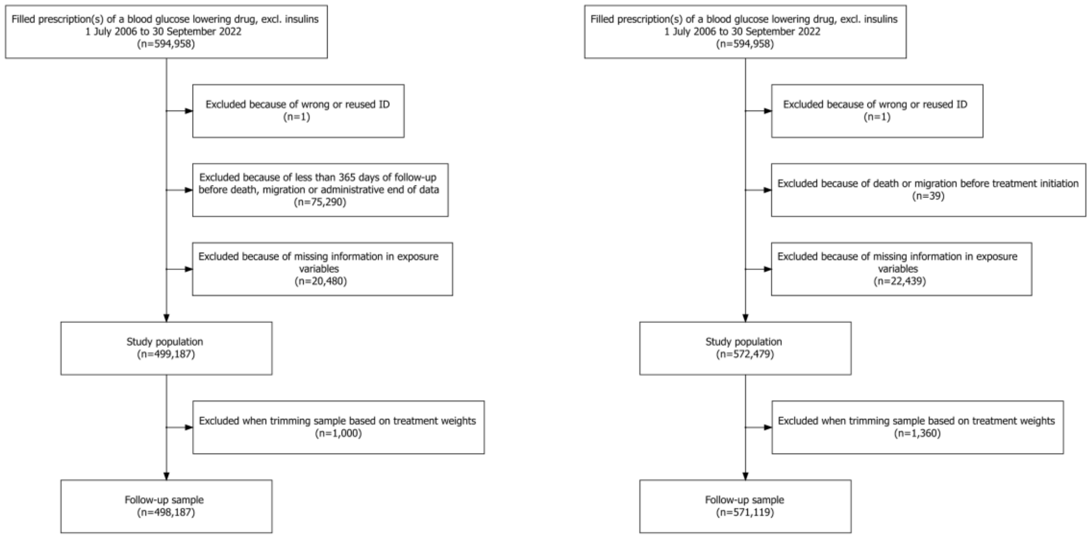

Flowchart for the study population presented to the left refers to the analysis for the outcome adherence to the antidiabetic medication regimens during the first year. Flowchart for the study population presented to the right refers to the analysis for the outcome time to first interruption of any antidiabetic medication from treatment initiation.

Table S1: Definitions of the exposure variables

Exposure variable	
Place of residence	Retrieved from the Total Population Register, categorized according to the classification provided by the Swedish Association of Local Authorities and Regions (SKR) as<br>i) **Large cities and municipalities near large cities.** Municipalities with a population of at least 200.000 inhabitants in the largest urban area, and municipalities where more than 40% of the working population commutes to work in a large city or municipality near a large city.<br>ii) **Medium-sized towns and municipalities near medium-sized towns.** Municipalities with a population of at least 50.000 inhabitants, with at least 40.000 inhabitants in the largest urban area, and commuting municipalities (including municipalities with a low commuting rate) near medium-sized towns.<br>iii) **Smaller towns/urban areas and rural municipalities.** Municipalities, and rural municipalities, with a population of less than 40.000 inhabitants in the largest urban area, and commuting municipalities near small towns.
Disposable income per consumption unit	Retrieved from the Longitudinal integrated database for health insurance and labour market studies (LISA). The total family income is weighted to be able to compare households of different sizes and compositions (the number of people is converted into a number of consumption units). Categorized depending on quartiles of disposable household income, from low (income quartile 1) to high income (income quartile 4).
Presence of income support	Retrieved from the Total Population Register. Categorized as having had any social income support or not during the calendar year.
Cohabitation status	Retrieved from the LISA. Categorized as cohabiting with someone, as married, registered partner, or having someone else contributing to the disposable family income, or not.

Table S2: Included antidiabetic medication, ATC codes, assumed daily doses and calculation of duration of the prescriptions

Drug class	Drug substance	ATC code	Assumed daily dose	Duration (days)
Insulins	Insulins	A10A		90
Biguanides	metformin	A10BA02	1 DDD per day	PACK DDD*NO
Combinations	*metformin and rosiglitazone*	*A10BD03*	1.5 tablets per day	(PACK SIZE*NO)/1.5
	metformin and glimepiride	*A10BD04*	1.5 tablets per day	(PACK SIZE*NO)/1.5
	metformin and pioglitazone	*A10BD05*	2 tablets per day	(PACK SIZE*NO)/2
	metformin and sitagliptin	*A10BD07*	1.5 tablets per day	(PACK SIZE*NO)/1.5
	metformin and vildagliptin	*A10BD08*	2 tablets per day	(PACK SIZE*NO)/2
	metformin and saxagliptin	*A10BD10*	2 tablets per day	(PACK SIZE*NO)/2
	metformin and linagliptin	*A10BD11*	2 tablets per day	(PACK SIZE*NO)/2
	metformin and dapagliflozin	*A10BD15*	2 tablets per day	(PACK SIZE*NO)/2
	metformin and canagliflozin	*A10BD16*	2 tablets per day	(PACK SIZE*NO)/2
	metformin and empagliflozin	*A10BD20*	2 tablets per day	(PACK SIZE*NO)/2
	metformin and ertugliflozin	*A10BD23*	2 tablets per day	(PACK SIZE*NO)/2
Sulfonylureas	glibenclamide	A10BB01	1 DDD per day	PACK DDD*NO
	glipizide	A10BB07	1 DDD per day	PACK DDD*NO
	glimepiride	A10BB12	1 tablet per day	PACK SIZE*NO
Combinations	*metformin and glimepiride*	*A10BD04*	1.5 tablets per day	(PACK SIZE*NO)/1.5
	glimepiride and pioglitazone	*A10BD06*	1 tablet per day	PACK SIZE*NO
DPP-4	sitagliptin	A10BH01	1 tablet per day	PACK SIZE*NO
	vildagliptin	A10BH02	2 tablets per day	(PACK SIZE*NO)/2
	saxagliptin	A10BH03	1 tablet per day	PACK SIZE*NO
	linagliptin	A10BH05	1 tablet per day	PACK SIZE*NO
Combinations	*metformin and sitagliptin*	*A10BD07*	1.5 tablets per day	(PACK SIZE*NO)/1.5
	metformin and vildagliptin	*A10BD08*	2 tablets per day	(PACK SIZE*NO)/2
	metformin and saxagliptin	*A10BD10*	2 tablets per day	(PACK SIZE*NO)/2
	metformin and linagliptin	*A10BD11*	2 tablets per day	(PACK SIZE*NO)/2
	linagliptin and empagliflozin	*A10BD19*	1 tablet per day	PACK SIZE*NO
	saxagliptin and dapagliflozin	*A10BD21*	1 tablet per day	PACK SIZE*NO
	sitagliptin and ertugliflozin	*A10BD24*	2 tablets per day	(PACK SIZE*NO)/2
GLP-1	exenatide	A10BJ01	1 DDD per day	PACK DDD*NO
	liraglutide (brand name Victoza; parenteral formulation with indication T2D)	A10BJ02	1.8 mg per day	30*NO
	lixisenatide	A10BJ03	1 dose per day	PACK SIZE*NO
	dulaglutide	A10BJ05	1 dose per week	(PACK SIZE*NO)*7
	semaglutide (brand name Ozempic; parenteral formulation with indication T2D)	A10BJ06	1 dose per week	(PACK SIZE*NO)*7
	semaglutide (brand name Rybelsus; oral formulation with indication T2D)	A10BJ06	1 tablet per day	PACK SIZE*NO
Combinations	*insulin, glargine and lixisenatide*	*A10AE54*		90
	insulin, degludek and liraglutide	*A10AE56*		90
SGLT2	dapagliflozin	A10BK01	1 tablet per day	PACK SIZE*NO
	canagliflozin	A10BK02	1 tablet per day	PACK SIZE*NO
	empagliflozin	A10BK03	1 tablet per day	PACK SIZE*NO
	ertugliflozin	A10BK04	1 tablet per day	PACK SIZE*NO
Combinations	*metformin and dapagliflozin*	*A10BD15*	2 tablets per day	(PACK SIZE*NO)/2
	metformin and canagliflozin	*A10BD16*	2 tablets per day	(PACK SIZE*NO)/2
	metformin and empagliflozin	*A10BD20*	2 tablets per day	(PACK SIZE*NO)/2
	metformin and ertugliflozin	*A10BD23*	2 tablets per day	(PACK SIZE*NO)/2
	linagliptin and empagliflozin	*A10BD19*	1 tablet per day	PACK SIZE*NO
	saxagliptin and dapagliflozin	*A10BD21*	1 tablet per day	PACK SIZE*NO
	sitagliptin and ertugliflozin	*A10BD24*	2 tablets per day	(PACK SIZE*NO)/2
Others	acarbose	A10BF01	6 tablets per day	(PACK SIZE*NO)/6
	rosiglitazone	A10BG02	1 DDD per day	PACK DDD*NO
	pioglitazone	A10BG03	1 tablet per day	PACK SIZE*NO
	repaglinide	A10BX02	1 DDD per day	PACK DDD*NO
	nateglinide	A10BX03	3 tablets per day	(PACK SIZE*NO)/3
Combinations	*metformin and pioglitazone*	*A10BD05*	2 tablets per day	(PACK SIZE*NO)/2
	metformin and rosiglitazone	*A10BD03*	1.5 tablets per day	(PACK SIZE*NO)/1.5
	glimepiride and pioglitazone	*A10BD06*	1 tablet per day	PACK SIZE*NO
	pioglitazone and alogliptin	*A10BD09*	1 tablet per day	PACK SIZE*NO

NO: number of packages that have been dispensed; PACK DDD: number of DDDs per package; PACK SIZE: pack size in numerical form.

Table S3: Definitions of the background covariates and confounding variables

Variable	Assessment period	ICD-10/ATC codes
Country of birth	Total Population Register, 2021	
Sweden		
The Nordic countries excl. Sweden		
Europe excl. E27 and the Nordic countries		
EU27 excl. the Nordic countries		
Asia		
Africa		
North America		
South America		
Oceania		
Unknown		
Age	The year prior measuring the exposures	
Migration	Total Population Register, 2021	
Sex	Total Population Register, 2021	
Education	Total Population Register, 2021	
Less than secondary education		
Secondary education		
More than secondary education		
Unknown		
Unemployed for more than 6 months	The year prior measuring the exposures	
No		
Yes		
Unknown		
Main income source	The year prior measuring the exposures	
Employment		
Unemployment		
Early retirement and social security		
Old age pensions		
None		
Unknown		
CCI score	The year prior measuring the exposures	
None (score of 0)		
Moderate (score of 1-4)		
Severe (score ≥5)		
Number of other filled prescriptions	The year prior measuring the exposures	Number of unique 5th level ATC codes
CVD	The year prior measuring the exposures	ICD-10 codes: I09, I11, I13, I20-25, I42-51, I70-79
Yes		
No		
Hypertension	The year prior measuring the exposures	ATC codes: C02-03, C07-09<br>ICD-10 codes: I10-15
Yes		
No		
Depression	The year prior measuring the exposures	ATC codes: N06A<br>ICD-10 codes: F32-39
Yes		
No		
Obesity	The year prior measuring the exposures	ATC codes: A08<br>ICD-10 code: E66
Yes		
No		
Number of inpatient care days	The year prior measuring the exposures	
None		
Low (number of days in hospital is >0 and ≤10)		
Moderate (number of days in hospital is >10 and ≤20)		
High (number of days in hospital is >20 and ≤50)		
Very high (number of days in hospital is >50)		

CCI: Charlson comorbidity index, CVD: cardiovascular diseases.

Figure S2: DAG representing relationships between outcome, exposures and background covariates, including confounders

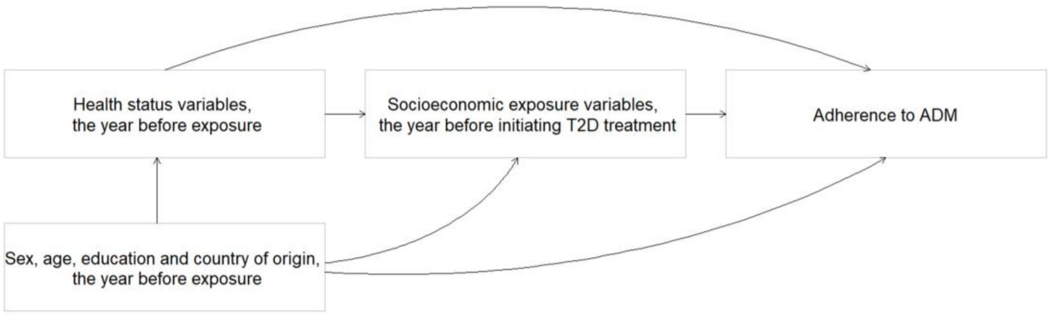

ADM: Antidiabetic medication; T2D: Type 2 Diabetes.

Table S4: The different comorbidity domains included in the Charlson comorbidity index (CCI) adapted to a Swedish setting

Domain	Included ICD-10 codes
Myocardial infarction	I21, I22, I25.2
Congestive heart failure	I11.0, I13.0, I13.2, I25.5, I42.0, I42.6, I42.7, I42.8, I42.9, I43, I50
Peripheral vascular disease	I70, I71, I731, I73.8, I73.9, I77.1, I79.0, I79.2, K55
Cerebrovascular disease	G45, I60, I61, I62, I63, I64, I67, I69
Chronic obstructive pulmonary disease (COPD)	J43, J44
Other chronic pulmonary disease	J41, J42, J45-47, J60-70
Rheumatic disease	M05, J06, J12.3, J07.0, J07.1, J07.2, J07.3, M08, M13, M30, M31.3, M31.4, M31.5, M31.6, M32-34, M35.0, M35.1, M35.3, M45, M46
Dementia<br>Hemiplegia tetraplegia	F00, F01, F02, F03, F05.1, G30, G31.1, G31.9<br>G11.4, G80, G81, G82, G83.0, G83.1, G83.2, G83.3, G83.8
Diabetes	E10.0, E10.1, E11.0, E11.1, E12.0, E12.1, E13.0, E13.1, E14.0, E14.1
Diabetes with end organ damage	E10.2, E10.3, E10.4, E10.5, E10.7, E11.2, E11.3, E11.4, E11.5<br>E11.6, E11.7, E12.2, E12.3, E12.4, E12.5, E12.6, E12.7, E13.2, E13.3, E13.4, E13.5, E13.6, E13.7, E14.2, E14.3, E14.4, E14.5, E14.6, E14.7
Moderate or severe kidney disease	I12.0, I13.1, N03.2, N03.3, N03.4, N03.5, N03.6, N03.7, N05.2, N05.3, N05.4, N05.5, N05.6, N05.7, N11, N18, N19, N25.0, Q61.1, Q61.2, Q61.3, Q61.4, Z49, Z94.0, Z99.2
Mild liver disease	B15, B16, B17, B18, B19, K70.3, K70.9, K73, K74.6, K75.4
Moderate or severe liver disease	I85.0, I85.9, I98.2, I98.3
(Peptic) Ulcer disease	K25, K26, K27, K28
Any malignancy including leukaemia and lymphoma	C00, C01-09, C10-41, C43, C45-58, C60-76, C81-86, C88-97
Metastatic cancer	C77, C78, C79, C80
HIV/AIDS	B20, B21, B22, B23, B24, F02.4, O98.7, R75, Z21.9, Z71.7

The different comorbidity domains included in the CCI adapted to a Swedish setting.[1]

Text S1: Subject-specific stabilized weight calculation

The outcome models for the outcome proportion of days covered by antidiabetic medication included all four exposure variables (A_j with $j = 1,2,3,4$) and the subject-specific stabilized treatment weight for individual $i = 1, \dots, n$, STW_i for fixed-time joint-exposures was

$$STW_i = STW_i^{A_1} \times STW_i^{A_2} \times STW_i^{A_3} \times STW_i^{A_4}$$

with

$$STW_i^{A_j} = \frac{P(A_{i,j})}{P(A_{i,j}|X_i)}$$

where X_i included both background covariates and potential confounders as well as potential interaction terms and higher moments for the continuous covariates.

For the outcome time to first treatment interruption, time-varying inverse probability of censoring weights (IPCW) were used. The risk of dying or being hospitalized, $P\left(C_{i,t}^{death,hosp} = 1 \middle| A_{i,j}, X_i\right)$, for each follow-up time point *t* was estimated with a logistic model, the stabilized censoring weight for individual i at time *t* was then

$$SCW_{i,t}^{A_j,death,hosp} = \frac{\prod_{k=1}^{t} 1 - P\left(C_{i,k}^{death,hosp} = 1 \middle| A_{i,j}\right)}{\prod_{k=1}^{t} 1 - P\left(C_{i,k}^{death,hosp} = 1 \middle| A_{i,j}, X_i\right)},$$

where X_i includes month after initiation, squared month after initiation, age, education, sex and CCI. The risk of migrating, $P\left(C_{i,t}^{mig} = 1 \middle| A_{i,j}, X_i\right)$, for each follow-up time point *t* was evaluated with a logistic model, and

$$SCW_{i,t}^{A_j,mig} = \frac{\prod_{k=1}^{t} 1 - P\left(C_{i,k}^{mig} = 1 \middle| A_{i,j}\right)}{\prod_{k=1}^{t} 1 - P\left(C_{i,k}^{mig} = 1 \middle| A_{i,j}, X_i\right)},$$

where X_i includes the year of treatment initiation, sex and country of birth. The risk of administrative end of study, $P\left(C_{i,t}^{cens} = 1 \middle| A_{i,j}, X_i\right)$, for each follow-up time point *t* was estimated with a logistic model, and

$$SCW_{i,t}^{A_j,cens} = \frac{\prod_{k=1}^{t} 1 - P\left(C_{i,k}^{cens} = 1 \middle| A_{i,j}\right)}{\prod_{k=1}^{t} 1 - P\left(C_{i,k}^{cens} = 1 \middle| A_{i,j}, X_i\right)},$$

where X_i includes the year of treatment initiation. The stabilized censoring weights were

$$SCW_{i,t}^{A_j} = SCW_{i,t}^{A_j,death,hosp} \times STCW_{i,t}^{A_j,mig} \times SCW_{i,t}^{A_j,cens}$$

and the subject-specific final stabilized weight for time *t* used to model the cause-specific risks of interruption was then

$$SW_{i,t}^{A_j} = STW_i^{A_j} \times SCW_{i,t}^{A_j}.$$

In the explorative analysis looking at relative risk of high versus low risk groups, the final weights for the joint exposure were

$$SW_{i,t} = SW_{i,t}^{A_1} \times SW_{i,t}^{A_2} \times SW_{i,t}^{A_3} \times SW_{i,t}^{A_4}.$$

The final stabilized weights were trimmed to include all individuals with final weights within the quantile range of 0.001 to 0.999 of the common distribution, and the weights were then re-estimated to improve covariate balance.

Figure S3: Mean absolute covariate balance at baseline, analysis of adherence measured with the PDC during the first year from treatment initiation

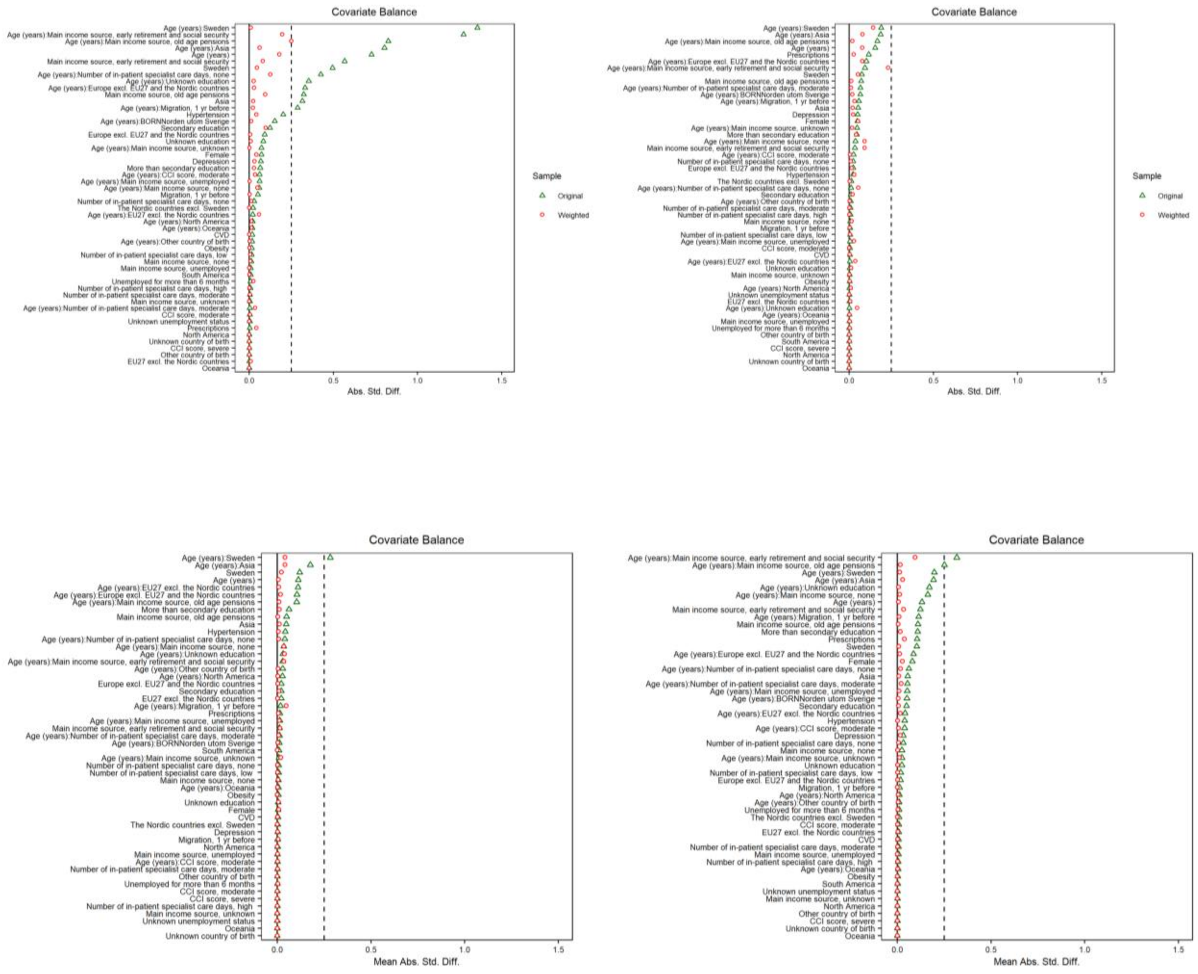

Absolute covariate balance at baseline for social income support (upper left panel), cohabitation (upper right panel), and mean absolute covariate balance at baseline for place of residence (lower left panel) and income (lower right panel). Green (Δ) is before and red (○) is after IPTW.

Table S5: Summary statistics of IPTW weights, analysis of adherence measured with the PDC during the first year from treatment initiation

	Min.	1st Qu.	Median	Mean	3rd Qu.	Max.
IPTW, STW_i	0.0042	0.4750	0.6936	1.1138	1.0604	780.9982
Trimmed	0.0145	0.4755	0.6936	1.0483	1.0590	31.4560
Trimmed re-weighted	0.0136	0.4746	0.6940	1.0598	1.0601	70.7608

Table S6: Summary statistics in the four outcome groups, analysis of adherence measured with the PDC during the first year from treatment initiation

Covariate	Non-adherent	Low adherence	Moderate adherence	High adherence
Age	56.681 (18.6159)	61.809 (14.8467)	60.902 (13.343)	59.802 (14.2382)
Secondary education	0.425 (0.4944)	0.442 (0.4966)	0.460 (0.4984)	0.462 (0.4986)
More than secondary education	0.248 (0.4318)	0.221 (0.4149)	0.213 (0.4097)	0.222 (0.4157)
Unknown education	0.033 (0.1796)	0.021 (0.1443)	0.019 (0.1360)	0.021 (0.1440)
CCI score of 1-4	0.061 (0.2398)	0.063 (0.2429)	0.060 (0.2378)	0.080 (0.2715)
CCI score of ≥5	0.002 (0.0438)	0.002 (0.0458)	0.002 (0.0469)	0.003 (0.0519)
Female	0.552 (0.4972)	0.467 (0.4989)	0.420 (0.4935)	0.401 (0.4900)
Number of other filled prescriptions	5.547 (5.4380)	5.738 (5.1126)	5.502 (5.2058)	5.654 (5.6548)
Number of days in hospital is >20 and ≤50	0.011 (0.1055)	0.010 (0.0986)	0.011 (0.1031)	0.013 (0.1127)
Number of days in hospital is >0 and ≤10	0.121 (0.3259)	0.104 (0.3058)	0.098 (0.2978)	0.099 (0.2989)
Number of days in hospital is >10 and ≤20	0.017 (0.1289)	0.015 (0.1216)	0.015 (0.1203)	0.019 (0.1351)
Number of days in hospital is zero	0.847 (0.3597)	0.867 (0.3397)	0.872 (0.3344)	0.864 (0.3431)

Weighted mean differences for the four outcome groups. Standard deviations within parentheses. CCI: Charlson comorbidity index.

Table S7: Subgroup analysis evaluating adherence via the PDC the first year after treatment initiation, subpopulation with a registered T2D diagnosis in the National Diabetes Register

		Non-adherent (PDC≤0.2) vs. high adherence (PDC>0.8)		Low adherence (0.2<PDC≤0.5) vs. high adherence (PDC>0.8)		Moderate adherence (0.5<PDC≤0.8) vs. high adherence (PDC>0.8)	
	N	aOR	95 % CI	aOR	95 % CI	aOR	95 % CI
Area of residence							
Large cities	103,036	1.23	1.19-1.28	0.98	0.96-1.01	0.90	0.88-0.92
Medium-sized towns	134,564	0.90	0.86-0.93	0.84	0.82-0.85	0.91	0.89-0.93
Smaller towns	93,661	ref		ref		ref	
Cohabitation							
Yes	206,137	1.03	0.99-1.06	1.08	1.05-1.10	1.06	1.04-1.08
No	125,124	ref		ref		ref	
Income							
Income quartile 1	72,272	1.25	1.19-1.30	0.99	0.96-1.02	1.08	1.04-1.11
Income quartile 2	85,539	1.23	1.18-1.28	1.03	1.00-1.06	1.08	1.05-1.11
Income quartile 3	86,256	1.12	1.07-1.17	0.98	0.95-1.01	1.04	1.01-1.07
Income quartile 4	87,194	ref		ref		ref	
Social income support							
Yes	17,566	1.31	1.24-1.39	0.98	0.94-1.02	0.99	0.95-1.04
No	313,695	ref		ref		ref	

N: total number of individuals per exposure group; aOR: adjusted odds ratio; CI: confidence interval; PDC: proportion of days covered.

Table S8. Exposure variables, and some background covariates and potential confounders for individuals excluded from the analysis of time to first treatment interruption of all antidiabetic medication

Exposure variables	
Place of residence	
– Large cities (%)	16.16
– Medium-sized towns (%)	18.10
– Small towns (%)	11.74
– Unknown (%)	54.00
Disposable family income per consumption unit	
– Mean and standard deviation	519.68 (1,388.29)
– Unknown (%)	60.97
Presence of income support	
– No (%)	26.02
– Yes (%)	13.01
– Unknown (%)	60.97
Cohabitation status	
– Yes (%)	29.34
– No (%)	9.69
– Unknown (%)	60.97
Background covariates and potential confounders	
Age (mean and standard deviation)	49.73 (16.90)
Migration (%)	2.11
Sex, Female (%)	46.04
County of birth	
– Sweden (%)	14.68
– The Nordic countries excl. Sweden (%)	5.27
– Europe excl. EU27 and the Nordic countries (%)	4.56
– EU27 excl. the Nordic countries (%)	6.47
– Asia (%)	48.91
– Africa (%)	14.70
– North America (%)	1.21
– South America (%)	1.67
– Oceania (%)	0.01
– Unknown (%)	2.28
Education	
– Less than secondary education (%)	0.53
– Secondary education (%)	0.55
– More than secondary education (%)	0.52
– Unknown (%)	98.39
Unemployment for more than 6 months	
– No (%)	1.69
– Yes (%)	0.08
– Unknown (%)	98.23
Main income source	
– Employment (%)	0.56

– Unemployment (%)	0.06
– Early retirement and social security (%)	0.43
– Old age pensions (%)	0.23
– None (%)	0.49
– Unknown (%)	98.23

EU27: European Union including the 27-member countries, income variables are in multiples of 100 SEK. Missing values in socioeconomic and demographic variables represent a missing value in the registers or the lack of a record in the register.

Table S9: Summary statistics of IPTW-IPCW weights, analysis of the outcome first interruption of all antidiabetic medication

		Min.	1st Qu.	Median	Mean	3rd Qu.	Max.
Social income support	Death/hospitalization	0.888	0.968	0.989	0.999	1.000	105.785
	Migration	0.993	0.999	1.000	1.000	1.000	1.041
	End of study	0.996	0.999	1.002	1.106	1.066	4.080
	IPTW, STW_i	0.061	0.948	0.952	1.002	0.971	95.982
	Final weight, $SW_{i,t}$	0.063	0.939	0.974	1.107	1.089	232.701
	Trimmed, re-weighted	0.072	0.941	0.975	1.098	1.091	99.379
Disposable income	Death/hospitalization	0.830	0.975	0.993	0.999	1.002	98.875
	Migration	0.992	0.999	1.000	1.000	1.000	1.036
	End of study	0.996	0.999	1.002	1.106	1.066	4.080
	IPTW, STW_i	0.252	0.594	0.758	1.027	1.045	121.530
	Final weight, $SW_{i,t}$	0.222	0.638	0.814	1.121	1.157	232.581
	Trimmed, re-weighted	0.222	0.637	0.813	1.106	1.156	221.862
Place of residence	Death/hospitalization	0.873	0.968	0.989	0.999	1.000	105.852
	Migration	0.994	0.999	1.000	1.000	1.000	1.038
	End of study	0.996	0.999	1.002	1.106	1.066	4.080
	IPTW, STW_i	0.355	0.855	0.964	1.000	1.066	9.281
	Final weight, $SW_{i,t}$	0.354	0.888	0.985	1.105	1.203	101.882
	Trimmed, re-weighted	0.418	0.889	0.986	1.105	1.202	103.122
Cohabitation	Death/hospitalization	0.846	0.971	0.991	0.999	1.001	100.689
	Migration	0.996	0.999	1.000	1.000	1.000	1.040
	End of study	0.996	0.999	1.002	1.106	1.066	4.080
	IPTW, STW_i	0.406	0.859	0.945	1.001	1.106	227.420
	Final weight, $SW_{i,t}$	0.380	0.883	0.997	1.102	1.193	226.813
	Trimmed, re-weighted	0.421	0.886	0.999	1.102	1.192	76.439

The numbers of trimmed individuals were 1,540 (social income support), 533 (disposable income), 959 (place of residence) and 1709 (cohabitation), respectively.

Figure S4: Mean absolute covariate balance at 12 months, analysis of the outcome first interruption of all antidiabetic medication

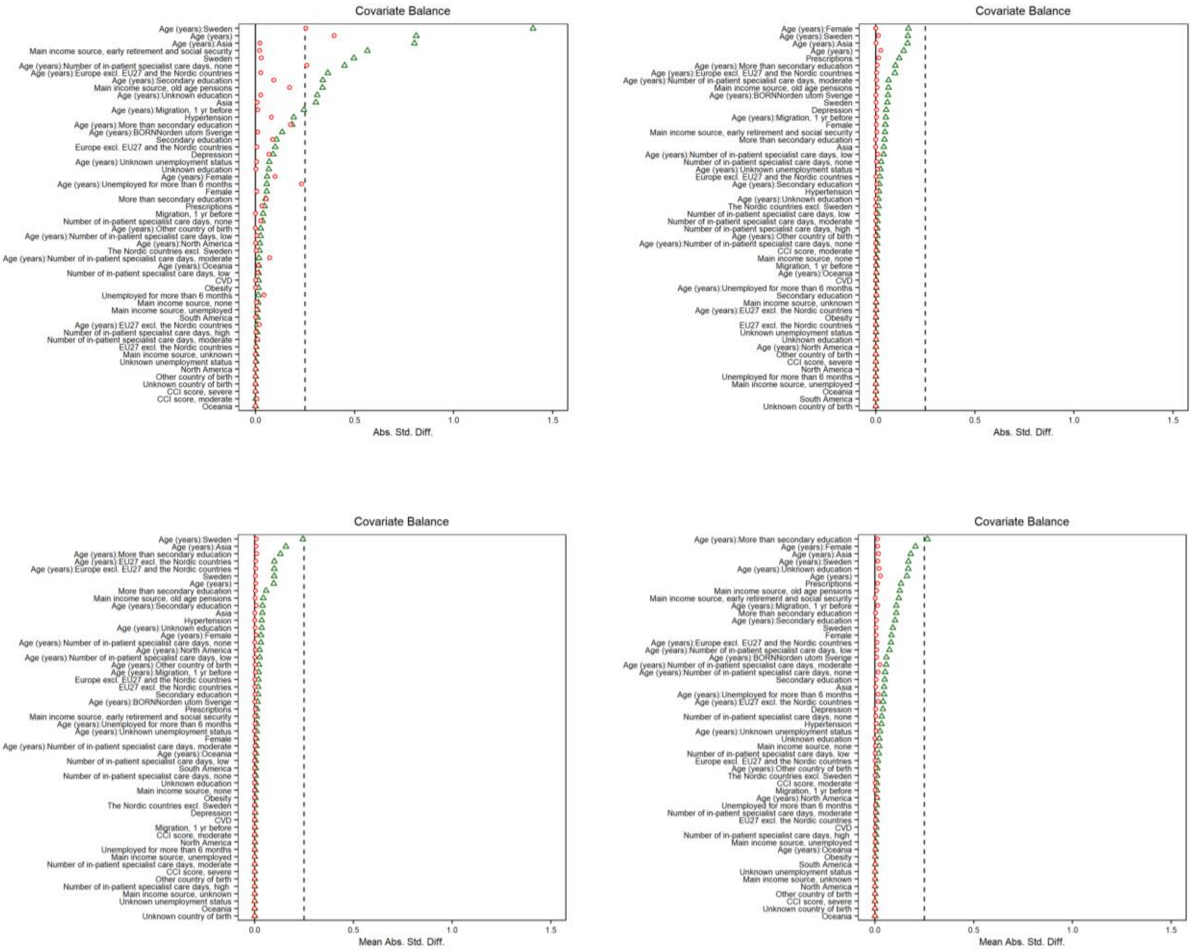

Absolute covariate balance at 12 months for social income support (upper left panel), cohabitation (upper right panel), and mean absolute covariate balance at 12 months for place of residence (lower left panel) and income (lower right panel). Green (Δ) is before and red (○) is after inverse probability of treatment and censoring weighting (IPTW-IPCW).

Figure S5: Mean absolute covariate balance at 60 months, analysis of the outcome first interruption of all antidiabetic medication

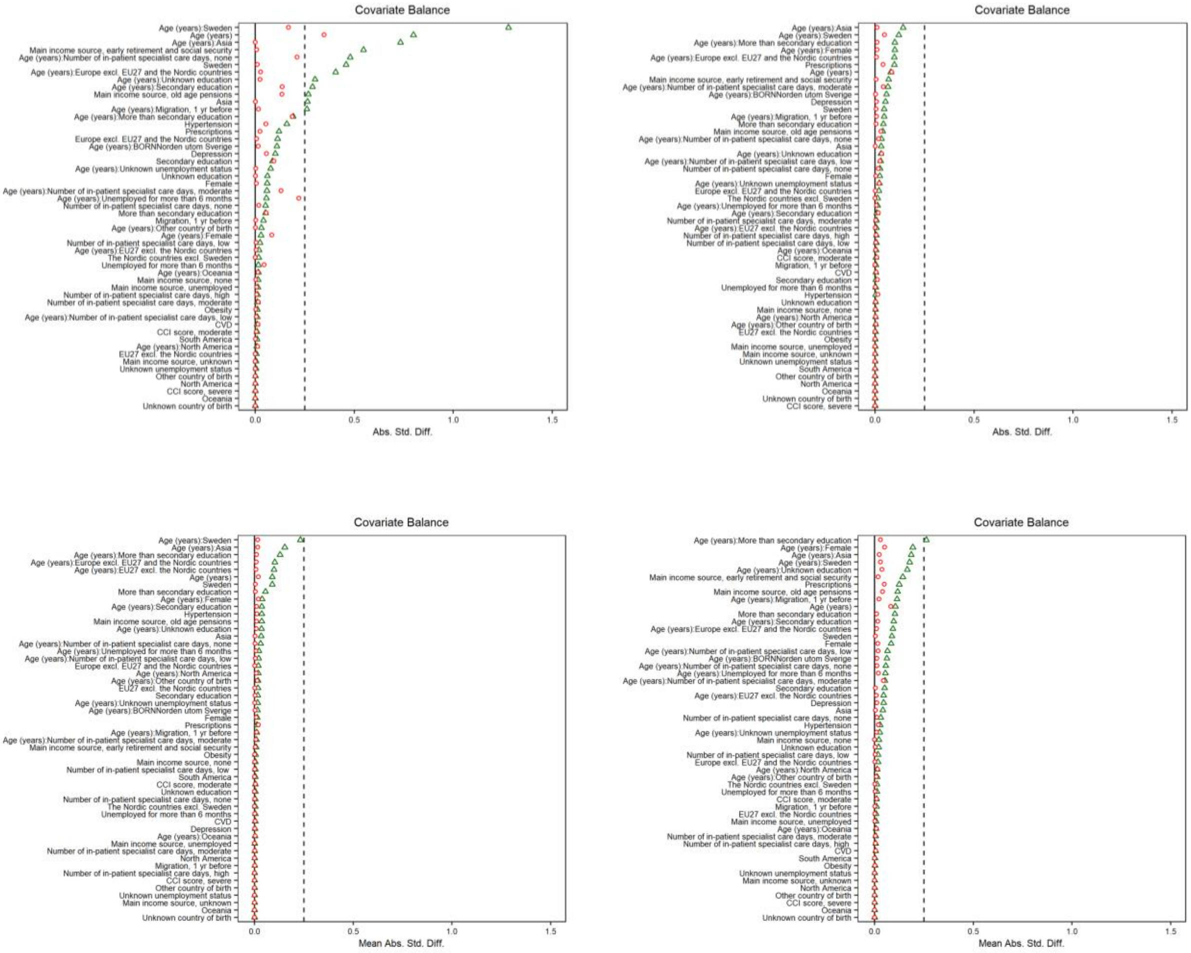

Absolute covariate balance at 60 months for social income support (upper left panel), cohabitation (upper right panel), and mean absolute covariate balance at 60 months for place of residence (lower left panel) and income (lower right panel). Green (Δ) is before and red (○) is after IPTW-IPCW.

References

1. Ludvigsson JF, Appelros P, Askling J, et al. Adaptation of the Charlson comorbidity index for register-based research in Sweden. *Clin Epidemiol.* 2021: **13**; 21–41.
